# Supplementary material for: Gut Bacteroides act in a microbial consortium to cause susceptibility to severe malaria
Source: Nat Commun. 2023 Oct 13;14:6465. doi: 10.1038/s41467-023-42235-0 (PMC10575898; doi:10.1038/s41467-023-42235-0)
Supplement: Supplementary file 1 — Supplementary Information [file 41467_2023_42235_MOESM1_ESM.pdf]

Gut *Bacteroides* act in a microbial consortium to cause susceptibility to severe malaria

Rabindra K. Mandal[1], Anita Mandal[1], Joshua E. Denny[2], Ruth Namazii[3], Chandy C. John[1], and Nathan W. Schmidt[1,2]*

[1]Ryan White Center for Pediatric Infectious Diseases and Global Health, Herman B Wells Center for Pediatric Research, Department of Pediatrics, Indiana University School of Medicine

[2]Department of Microbiology and Immunology, University of Louisville

[3]Department of Paediatrics and Child Health, Makerere University, Kampala, Uganda

Supplementary Information

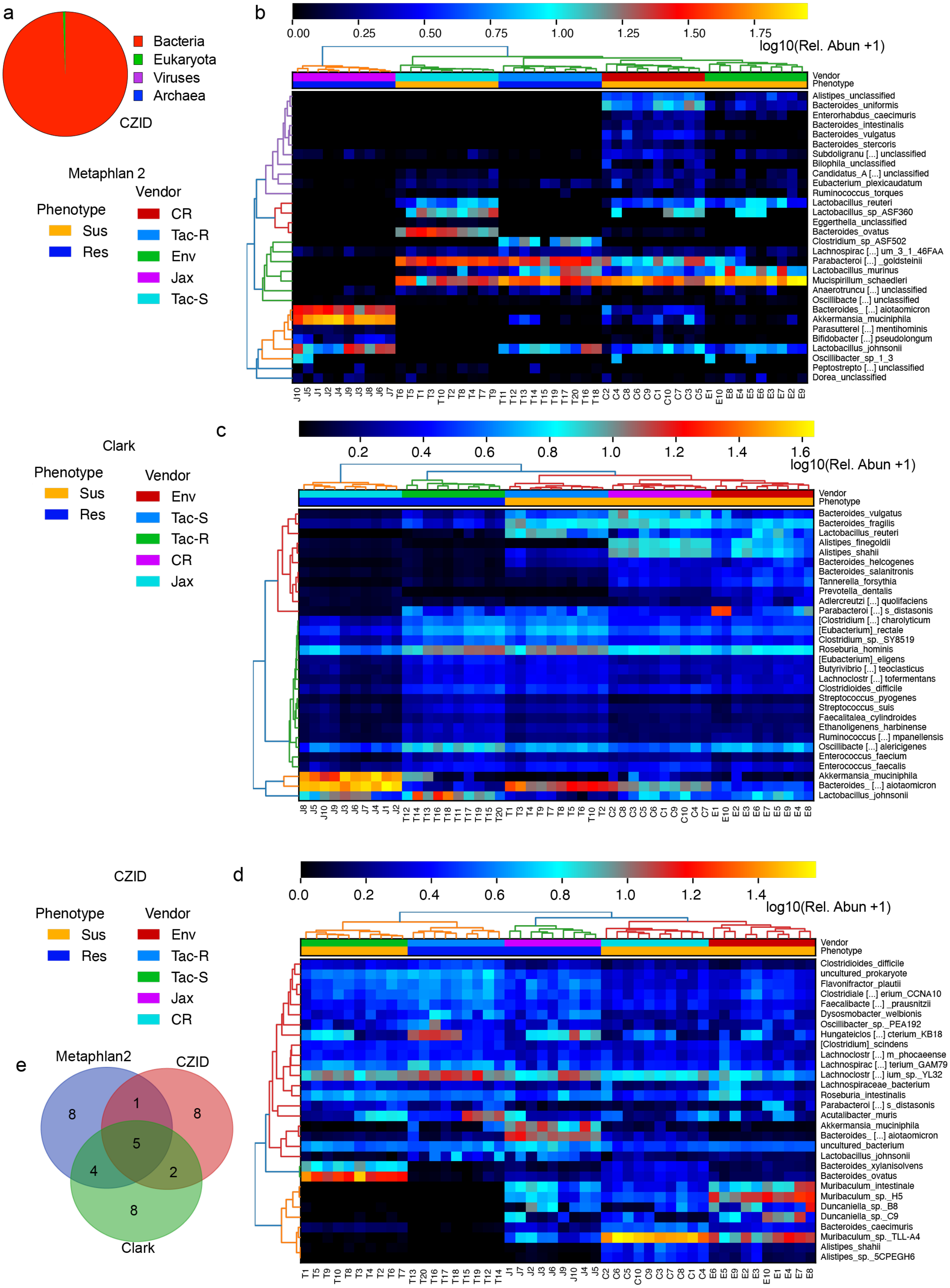

a
Bacteria
Eukaryota
Viruses
Archaea
CZID
Metaphlan 2
Phenotype
Sus
Res
Vendor
CR
Tac-R
Env
Jax
Tac-S
b
0.00 0.25 0.50 0.75 1.00 1.25 1.50 1.75
log10(Rel. Abun +1)
Vendor
Phenotype
Alistipes_unclassified
Bacteroides_uniformis
Enterorhabdus_caecimuris
Bacteroides_intestinalis
Bacteroides_vulgatus
Bacteroides_stercoris
Subdoligranu [...] unclassified
Bilophila_unclassified
Candidatus_A [...] unclassified
Eubacterium_plexicaudatum
Ruminococcus_torques
Lactobacillus_reuteri
Lactobacillus_sp_ASF360
Eggerthella_unclassified
Bacteroides_ovatus
Clostridium_sp_ASF502
Lachnospirac [...] um_3_1_46FAA
Parabacteroi [...] _goldsteinii
Lactobacillus_murinus
Mucispirillum_schaedleri
Anaerotruncu [...] unclassified
Oscillibacte [...] unclassified
Bacteroides_ [...] aiotaomicron
Akkermansia_muciniphila
Parasutterel [...] mentihominis
Bifidobacter [...] pseudolongum
Lactobacillus_johnsonii
Oscillibacter_sp_1_3
Peptostrepto [...] unclassified
Dorea_unclassified
Clark
Phenotype
Sus
Res
Vendor
Env
Tac-S
Tac-R
CR
Jax
c
0.2 0.4 0.6 0.8 1.0 1.2 1.4 1.6
log10(Rel. Abun +1)
Vendor
Phenotype
Bacteroides_vulgatus
Bacteroides_fragilis
Lactobacillus_reuteri
Alistipes_finegoldii
Alistipes_shahii
Bacteroides_helcogenes
Bacteroides_salanitronis
Tannerella_forsythia
Prevotella_dentalis
Adlercreutzi [...] quolifaciens
Parabacteroi [...] s_distasonis
[Clostridium [...] charolyticum
[Eubacterium]_rectale
Clostridium_sp._SY8519
Roseburia_hominis
[Eubacterium]_eligens
Butyrivibrio [...] teoclasticus
Lachnoclostr [...] tofermentans
Clostridioides_difficile
Streptococcus_pyogenes
Streptococcus_suis
Faecalitalea_cylindroides
Ethanoligenens_harbinense
Ruminococcus [...] mpanellensis
Oscillibacte [...] alericigenes
Enterococcus_faecium
Enterococcus_faecalis
Akkermansia_muciniphila
Bacteroides_ [...] aiotaomicron
Lactobacillus_johnsonii
CZID
Phenotype
Sus
Res
Vendor
Env
Tac-R
Tac-S
Jax
CR
d
0.0 0.2 0.4 0.6 0.8 1.0 1.2 1.4
log10(Rel. Abun +1)
Vendor
Phenotype
Clostridioides_difficile
uncultured_prokaryote
Flavonifractor_plautii
Clostridiale [...] erium_CCNA10
Faecalibacte [...] _prausnitzii
Dysosmobacter_welbionis
Oscillibacter_sp._PEA192
Hungateiclos [...] cterium_KB18
[Clostridium]_scindens
Lachnoclostr [...] m_phocaeense
Lachnospirac [...] terium_GAM79
Lachnoclostr [...] ium_sp._YL32
Lachnospiraceae_bacterium
Roseburia_intestinalis
Parabacteroi [...] s_distasonis
Acutalibacter_muris
Akkermansia_muciniphila
Bacteroides_ [...] aiotaomicron
uncultured_bacterium
Lactobacillus_johnsonii
Bacteroides_xylanisolvens
Bacteroides_ovatus
Muribaculum_intestinale
Muribaculum_sp._H5
Duncaniella_sp._B8
Duncaniella_sp._C9
Bacteroides_caecimuris
Muribaculum_sp._TLL-A4
Alistipes_shahii
Alistipes_sp._5CPEGH6
e
Metaphlan2
CZID
Clark
8
1
8
5
4
2
8

S Figure 1 (Related to Figure 1): Taxonomic profile of naïve mice differentially susceptible to Py hyperparasitemia. Heatmap shows relative abundance of top 30 bacterial species detected using different bioinformatic approaches. **a** Microbiota detected at kingdom level using CZID. **b** Heatmap based on Metaphlan2 output. **c** Heatmap based on Clark output. **d** Heatmap based on CZID. **e** Venn diagram showing overlap at species level between the different bioinformatics approaches. Source data are provided in the Source Data file.

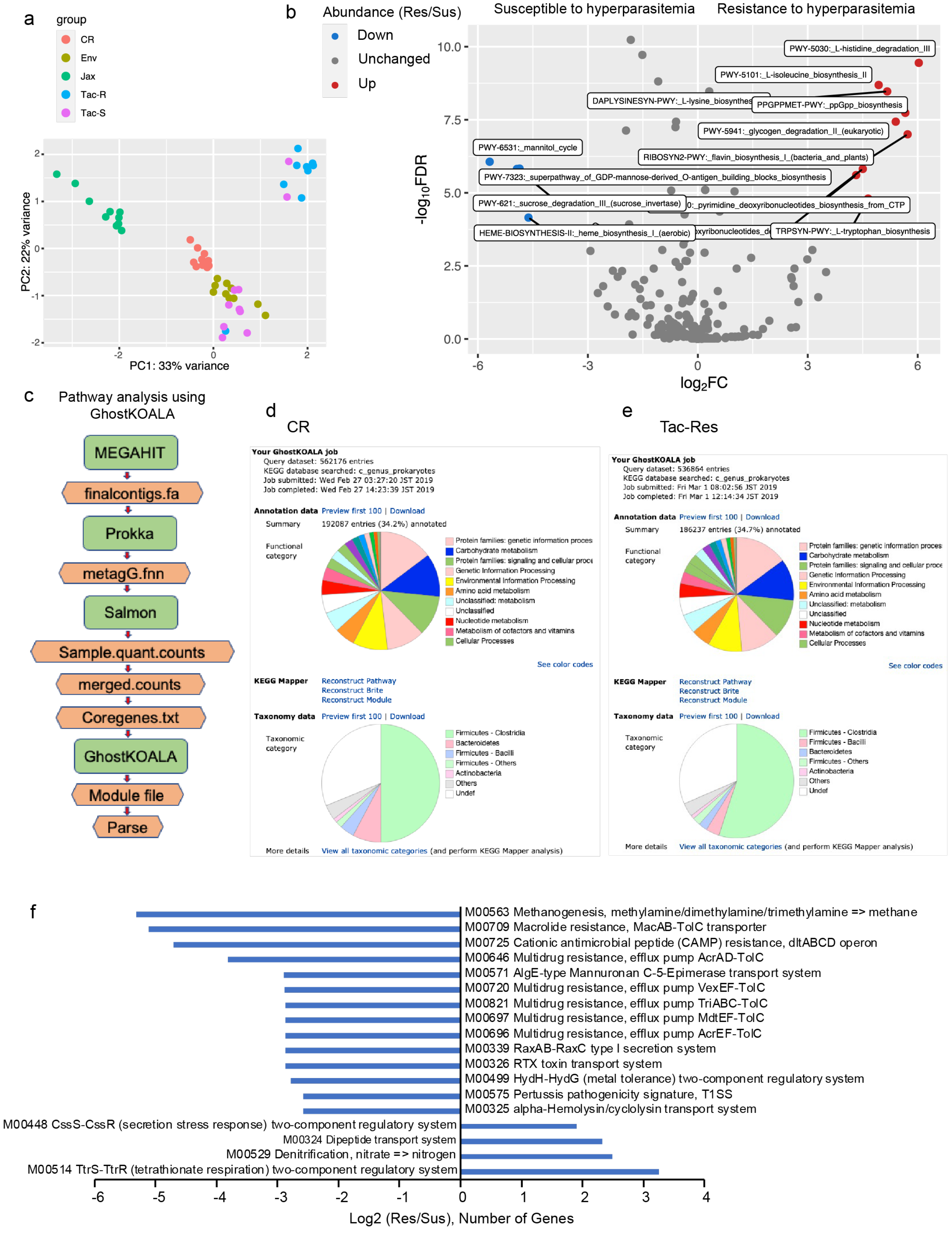

a
group
CR
Env
Jax
Tac-R
Tac-S
PC2: 22% variance
PC1: 33% variance
b
Abundance (Res/Sus)
Down
Unchanged
Up
Susceptible to hyperparasitemia
Resistance to hyperparasitemia
-log10FDR
log2FC
PWY-5030:_L-histidine_degradation_III
PWY-5101:_L-isoleucine_biosynthesis_II
DAPLYSINESYN-PWY:_L-lysine_biosynthes
PPGPPMET-PWY:_ppGpp_biosynthesis
PWY-5941:_glycogen_degradation_II_(eukaryotic)
PWY-6531:_mannitol_cycle
RIBOSYN2-PWY:_flavin_biosynthesis_I_(bacteria_and_plants)
PWY-7323:_superpathway_of_GDP-mannose-derived_O-antigen_building_blocks_biosynthesis
PWY-621:_sucrose_degradation_III_(sucrose_invertase)
0:_pyrimidine_deoxyribonucleotides_biosynthesis_from_CTP
HEME-BIOSYNTHESIS-II:_heme_biosynthesis_I_(aerobic)
TRPSYN-PWY:_L-tryptophan_biosynthesis
c
Pathway analysis using GhostKOALA
MEGAHIT
finalcontigs.fa
Prokka
metagG.fnn
Salmon
Sample.quant.counts
merged.counts
Coregenes.txt
GhostKOALA
Module file
Parse
d
CR
Your GhostKOALA job
Query dataset: 562176 entries
KEGG database searched: c_genus_prokaryotes
Job submitted: Wed Feb 27 03:27:20 JST 2019
Job completed: Wed Feb 27 14:23:39 JST 2019
Annotation data Preview first 100 | Download
Summary 192087 entries (34.2%) annotated
Functional category
Protein families: genetic information proces
Carbohydrate metabolism
Protein families: signaling and cellular proce
Genetic Information Processing
Environmental Information Processing
Amino acid metabolism
Unclassified: metabolism
Unclassified
Nucleotide metabolism
Metabolism of cofactors and vitamins
Cellular Processes
See color codes
KEGG Mapper Reconstruct Pathway
Reconstruct Brite
Reconstruct Module
Taxonomy data Preview first 100 | Download
Taxonomic category
Firmicutes - Clostridia
Bacteroidetes
Firmicutes - Bacilli
Firmicutes - Others
Actinobacteria
Others
Undef
More details View all taxonomic categories (and perform KEGG Mapper analysis)
e
Tac-Res
Your GhostKOALA job
Query dataset: 536864 entries
KEGG database searched: c_genus_prokaryotes
Job submitted: Fri Mar 1 08:02:56 JST 2019
Job completed: Fri Mar 1 12:14:34 JST 2019
Annotation data Preview first 100 | Download
Summary 186237 entries (34.7%) annotated
Functional category
Protein families: genetic information proces
Carbohydrate metabolism
Protein families: signaling and cellular proce
Genetic Information Processing
Environmental Information Processing
Amino acid metabolism
Unclassified: metabolism
Unclassified
Nucleotide metabolism
Metabolism of cofactors and vitamins
Cellular Processes
See color codes
KEGG Mapper Reconstruct Pathway
Reconstruct Brite
Reconstruct Module
Taxonomy data Preview first 100 | Download
Taxonomic category
Firmicutes - Clostridia
Firmicutes - Bacilli
Bacteroidetes
Firmicutes - Others
Actinobacteria
Others
Undef
More details View all taxonomic categories (and perform KEGG Mapper analysis)
f
M00563 Methanogenesis, methylamine/dimethylamine/trimethylamine => methane
M00709 Macrolide resistance, MacAB-TolC transporter
M00725 Cationic antimicrobial peptide (CAMP) resistance, dltABCD operon
M00646 Multidrug resistance, efflux pump AcrAD-TolC
M00571 AlgE-type Mannuronan C-5-Epimerase transport system
M00720 Multidrug resistance, efflux pump VexEF-TolC
M00821 Multidrug resistance, efflux pump TriABC-TolC
M00697 Multidrug resistance, efflux pump MdtEF-TolC
M00696 Multidrug resistance, efflux pump AcrEF-TolC
M00339 RaxAB-RaxC type I secretion system
M00326 RTX toxin transport system
M00499 HydH-HydG (metal tolerance) two-component regulatory system
M00575 Pertussis pathogenicity signature, T1SS
M00325 alpha-Hemolysin/cyclolysin transport system
M00448 CssS-CssR (secretion stress response) two-component regulatory system
M00324 Dipeptide transport system
M00529 Denitrification, nitrate => nitrogen
M00514 TtrS-TtrR (tetrathionate respiration) two-component regulatory system
Log2 (Res/Sus), Number of Genes

S Figure 2 (Related to Figure 1): Microbial pathway analysis in naïve mice differentially susceptible to Py hyperparasitemia. **a** Principal component analysis plot using HUMAnN 2 at pathway level. Volcano plot showing differentially abundant microbial pathways with log2FC > |3| and FDR <0.05. **b** Schematic showing pathway analysis using GhostKOALA. Green box represents bioinformatics package and orange represents the output file. **c** Screenshot of GhostKOALA output of Py hyperparasitemia susceptible CR mice. **d** Screenshot of GhostKOALA output of Py hyperparasitemia resistant Tac mice. **e** Pathways differentially abundant in Py hyperparasitemia susceptible (CR+Env) and resistant (Tac+Jax) mice. Log2FC is based on the number of microbial genes belonging to a given KEGG module. Source data are provided in the Source Data file.

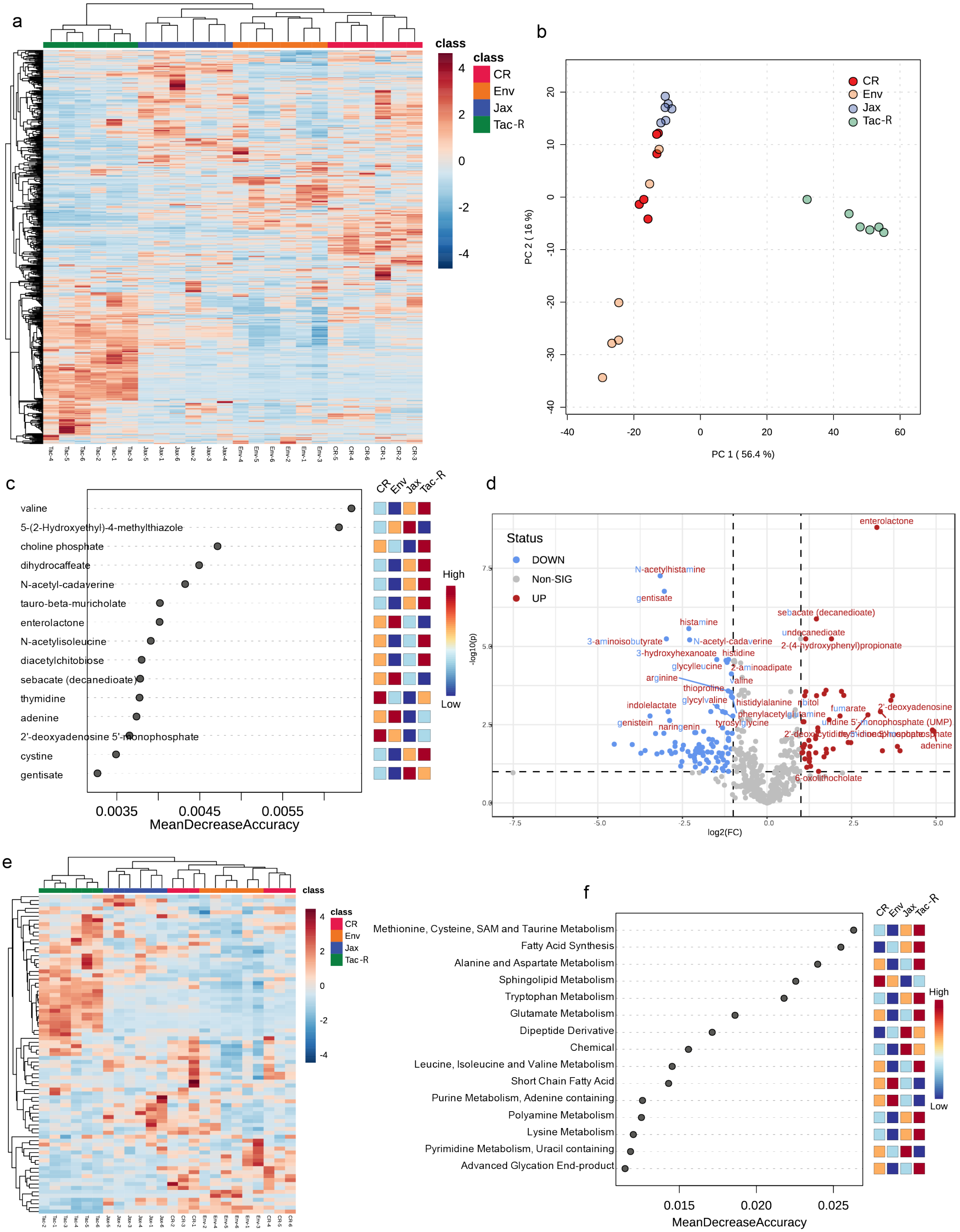
a
class
4
2
0
-2
-4
class
CR
Env
Jax
Tac-R
Tac-4 Tac-5 Tac-6 Tac-2 Tac-1 Tac-3 Jax-5 Jax-1 Jax-6 Jax-2 Jax-3 Jax-4 Env-4 Env-5 Env-6 Env-2 Env-1 Env-3 CR-5 CR-4 CR-6 CR-1 CR-2 CR-3
b
CR
Env
Jax
Tac-R
PC 2 (16 %)
PC 1 (56.4 %)
c
CR Env Jax Tac-R
valine
5-(2-Hydroxyethyl)-4-methylthiazole
choline phosphate
dihydrocaffeate
N-acetyl-cadaverine
tauro-beta-muricholate
enterolactone
N-acetylisoleucine
diacetylchitobiose
sebacate (decanedioate)
thymidine
adenine
2'-deoxyadenosine 5'-monophosphate
cystine
gentisate
High
Low
0.0035
0.0045
0.0055
MeanDecreaseAccuracy
d
Status
DOWN
Non-SIG
UP
enterolactone
N-acetylhistamine
gentisate
histamine
sebacate (decanedioate)
undecanedioate
3-aminoisobutyrate
N-acetyl-cadaverine
2-(4-hydroxyphenyl)propionate
3-hydroxyhexanoate
histidine
glycylleucine
2-aminoadipate
arginine
valine
thioproline
glycylvaline
histidylalanine
ribitol
indolelactate
phenylacetylglutamine
fumarate
2'-deoxyadenosine
genistein
tyrosylglycine
uridine 5'-monophosphate (UMP)
naringenin
adenine
6-oxolithocholate
-log10(p)
log2(FC)
e
class
class
CR
Env
Jax
Tac-R
f
CR Env Jax Tac-R
Methionine, Cysteine, SAM and Taurine Metabolism
Fatty Acid Synthesis
Alanine and Aspartate Metabolism
Sphingolipid Metabolism
Tryptophan Metabolism
Glutamate Metabolism
Dipeptide Derivative
Chemical
Leucine, Isoleucine and Valine Metabolism
Short Chain Fatty Acid
Purine Metabolism, Adenine containing
Polyamine Metabolism
Lysine Metabolism
Pyrimidine Metabolism, Uracil containing
Advanced Glycation End-product
High
Low
0.015
0.020
0.025
MeanDecreaseAccuracy

S Figure 3 (Related to Figure 1): Ceca metabolomics. Ceca contents were collected from hyperparasitemia susceptible (CR and Env) and resistant naïve mice (Tac-R and Jax) n=6/group. Global metabolomics were performed using LC-MS/MS. **a** Heatmap showing clustering of samples and metabolites. **b** Principal component analysis (PCA) plot showing clustering of samples by groups. **c** Features (metabolites) ranked by their contribution to classification accuracy (Mean Decrease Accuracy) among four groups using random forest classifier. **d** Volcano plot showing differentially abundant metabolites (Sus/Res). Metabolites with higher abundance in susceptible group are shown in red and in resistant group in blue with log2FC >|2| and false discovery rate < 0.05. **e** Heatmap showing clustering of samples and sub-pathway. **f** Features (sub-pathway) ranked by their contribution to classification accuracy (Mean Decrease Accuracy) among four groups using random forest classifier. Source data are provided in the Source Data file.

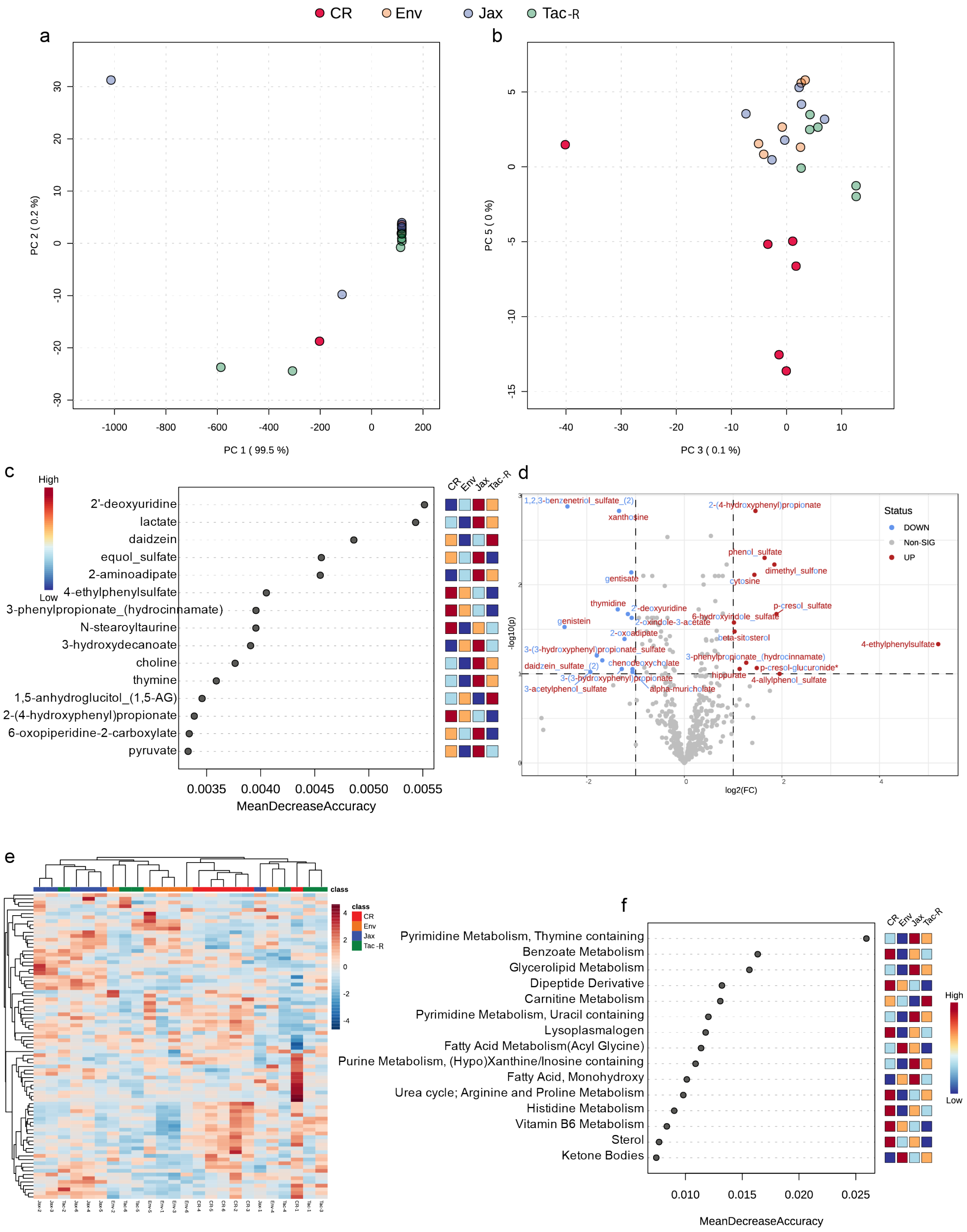
CR
Env
Jax
Tac-R
a
b
PC 1 (99.5 %)
PC 2 (0.2 %)
PC 3 (0.1 %)
PC 5 (0 %)
c
High
Low
2'-deoxyuridine
lactate
daidzein
equol_sulfate
2-aminoadipate
4-ethylphenylsulfate
3-phenylpropionate_(hydrocinnamate)
N-stearoyltaurine
3-hydroxydecanoate
choline
thymine
1,5-anhydroglucitol_(1,5-AG)
2-(4-hydroxyphenyl)propionate
6-oxopiperidine-2-carboxylate
pyruvate
MeanDecreaseAccuracy
d
Status
DOWN
Non-SIG
UP
1,2,3-benzenetriol_sulfate_(2)
xanthosine
2-(4-hydroxyphenyl)propionate
phenol_sulfate
dimethyl_sulfone
gentisate
cytosine
thymidine
2'-deoxyuridine
p-cresol_sulfate
6-hydroxyindole_sulfate
genistein
2-oxindole-3-acetate
2-oxoadipate
beta-sitosterol
3-(3-hydroxyphenyl)propionate_sulfate
4-ethylphenylsulfate
3-phenylpropionate_(hydrocinnamate)
daidzein_sulfate_(2)
chenodeoxycholate
p-cresol-glucuronide*
3-(3-hydroxyphenyl)propionate
hippurate
4-allylphenol_sulfate
3-acetylphenol_sulfate
alpha-muricholate
-log10(p)
log2(FC)
e
class
CR
Env
Jax
Tac-R
f
Pyrimidine Metabolism, Thymine containing
Benzoate Metabolism
Glycerolipid Metabolism
Dipeptide Derivative
Carnitine Metabolism
Pyrimidine Metabolism, Uracil containing
Lysoplasmalogen
Fatty Acid Metabolism(Acyl Glycine)
Purine Metabolism, (Hypo)Xanthine/Inosine containing
Fatty Acid, Monohydroxy
Urea cycle; Arginine and Proline Metabolism
Histidine Metabolism
Vitamin B6 Metabolism
Sterol
Ketone Bodies
High
Low
MeanDecreaseAccuracy

S Figure 4 (Related to Figure 1): Serum metabolomics. Sera were extracted from hyperparasitemia susceptible (CR and Env) and resistant naïve mice (Tac-R and Jax) n=6/group. Global metabolomics were performed using LC-MS/MS. **a** PCA plot showing clustering of samples by groups using PC1 and PC2. To note, 99.7% of the variation is explained by only 5 samples out of 24 samples. **b** PCA plot showing clustering of samples by groups using PC3 and PC5 where only 0.1% of variation is explained. **c** Features (metabolites) ranked by their contribution to classification accuracy (Mean Decrease Accuracy) among four groups using random forest classifier. **d** Volcano plot showing differentially abundant metabolites (Sus/Res) in sera. Metabolites with higher abundance in susceptible group are shown in red and in resistant group in blue with log2FC >|2| and false discovery rate < 0.05. **e** Heatmap showing clustering of samples and sub-pathway. **f** Features (sub-pathway) ranked by their contribution to classification accuracy (Mean Decrease Accuracy) among four groups using random forest classifier. Source data are provided in the Source Data file.

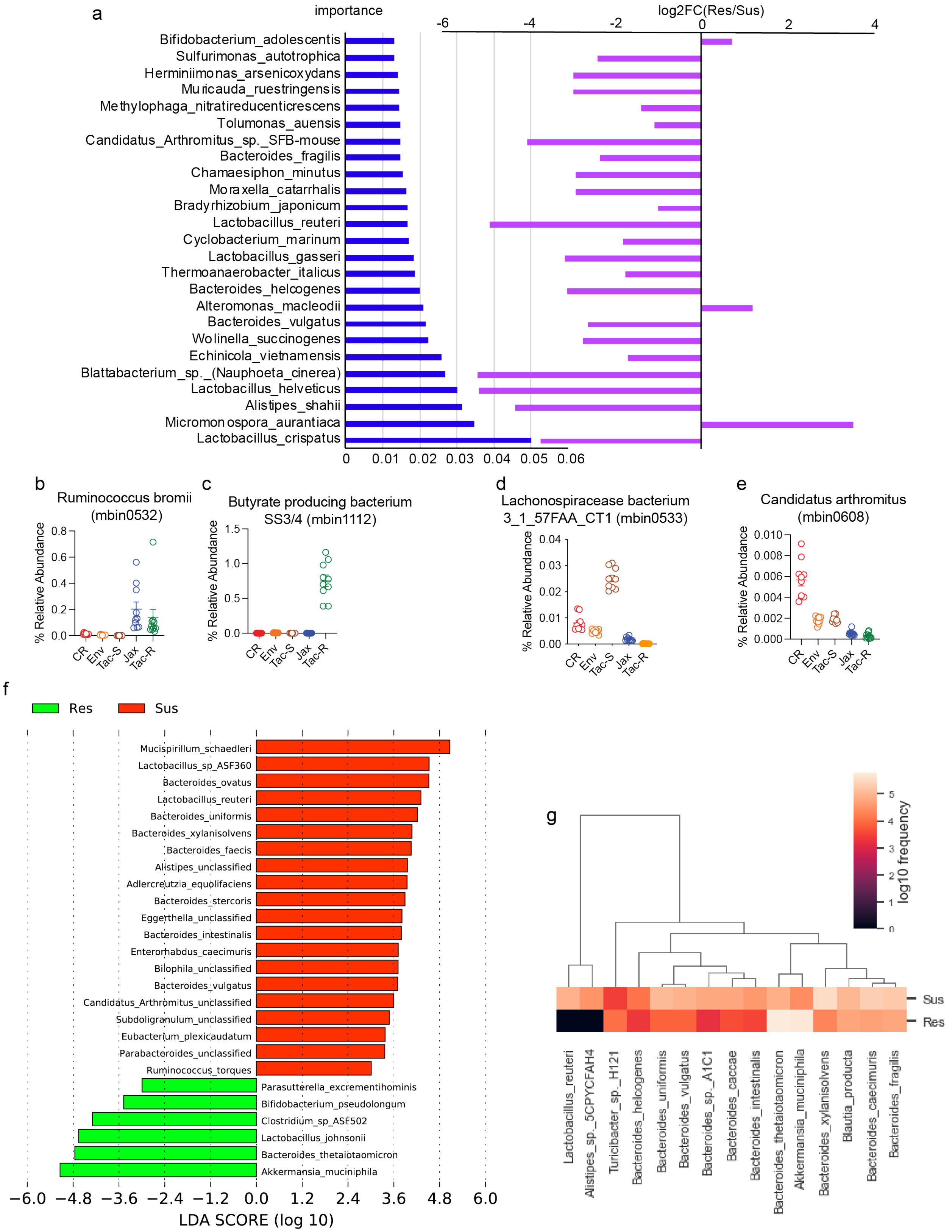

a
importance
log2FC(Res/Sus)
Bifidobacterium_adolescentis
Sulfurimonas_autotrophica
Herminiimonas_arsenicoxydans
Muricauda_ruestringensis
Methylophaga_nitratireducenticrescens
Tolumonas_auensis
Candidatus_Arthromitus_sp._SFB-mouse
Bacteroides_fragilis
Chamaesiphon_minutus
Moraxella_catarrhalis
Bradyrhizobium_japonicum
Lactobacillus_reuteri
Cyclobacterium_marinum
Lactobacillus_gasseri
Thermoanaerobacter_italicus
Bacteroides_helcogenes
Alteromonas_macleodii
Bacteroides_vulgatus
Wolinella_succinogenes
Echinicola_vietnamensis
Blattabacterium_sp._(Nauphoeta_cinerea)
Lactobacillus_helveticus
Alistipes_shahii
Micromonospora_aurantiaca
Lactobacillus_crispatus
b
Ruminococcus bromii (mbin0532)
c
Butyrate producing bacterium SS3/4 (mbin1112)
d
Lachnospiracease bacterium 3_1_57FAA_CT1 (mbin0533)
e
Candidatus arthromitus (mbin0608)
% Relative Abundance
CR Env Tac-S Jax Tac-R
f
Res
Sus
Mucispirillum_schaedleri
Lactobacillus_sp_ASF360
Bacteroides_ovatus
Lactobacillus_reuteri
Bacteroides_uniformis
Bacteroides_xylanisolvens
Bacteroides_faecis
Alistipes_unclassified
Adlercreutzia_equolifaciens
Bacteroides_stercoris
Eggerthella_unclassified
Bacteroides_intestinalis
Enterorhabdus_caecimuris
Bilophila_unclassified
Bacteroides_vulgatus
Candidatus_Arthromitus_unclassified
Subdoligranulum_unclassified
Eubacterium_plexicaudatum
Parabacteroides_unclassified
Ruminococcus_torques
Parasutterella_excrementihominis
Bifidobacterium_pseudolongum
Clostridium_sp_ASF502
Lactobacillus_johnsonii
Bacteroides_thetaiotaomicron
Akkermansia_muciniphila
LDA SCORE (log 10)
g
log10 frequency
Sus
Res
Lactobacillus_reuteri
Alistipes_sp._5CPYCFAH4
Turicibacter_sp._H121
Bacteroides_helcogenes
Bacteroides_uniformis
Bacteroides_vulgatus
Bacteroides_sp._A1C1
Bacteroides_caccae
Bacteroides_intestinalis
Bacteroides_thetaiotaomicron
Akkermansia_muciniphila
Bacteroides_xylanisolvens
Blautia_producta
Bacteroides_caecimuris
Bacteroides_fragilis

S Figure 5 (Related to Figure 2): Differential abundance analysis of gut bacterial species in naïve mice differentially susceptible to Py hyperparasitemia. a Importance of bacterial features able to classify samples based on malaria severity phenotype. Clark output at species level was fed to random forest classifier. Log2FC indicates directionality of bacterial abundance in Py hyperparasitemia resistant or susceptible mice. **b** Percent relative abundance of mbin0532 classified as *Ruminococcus bromii*. **c** Percent relative abundance of mbin1112 classified as Butyrate producing bacterium SS3/4. **d** Percent relative abundance of mbin0533 classified as *Lachonospiracease bacterium* 3_1_F7FAA_CT1. **e** Percent relative abundance of mbin0608 classified as *Candidatus arthromitus*. **f** LEfSe analysis showing differentially abundant species identified by MetaPhlan2. LDA score cutoff were >|3.5|. **g** Heatmap showing the top bacterial features able to classify samples to Py hyperparasitemia resistance or susceptible mice using CZID. Features were identified using random forest. All data are mean ± SE (standard error). Source data are provided in the Source Data file.

a

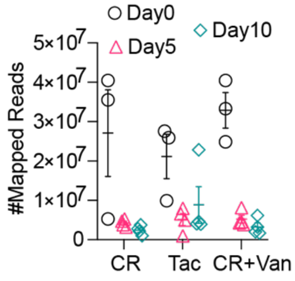

b

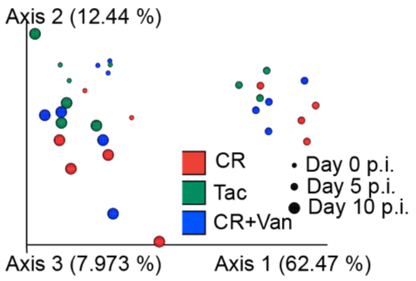

c

log2FC >= |2|

Group Camparision	pvalue(<0.05)	padj (<0.05)
D0-CR-vs-CR+Van	141	4
D0-CR-vs-Tac-R	151	2
D0-CR+Van-vs-Tac-R	129	1
D5-CR-vs-CR+Van	330	12
D5-CR-vs-Tac-R	321	14
D5-CR+Van-vs-Tac-R	300	0
D10-CR-vs-CR+Van	353	17
D10-CR-vs-Tac-R	496	12
D10-CR+Van-vs-Tac-R	288	3

d

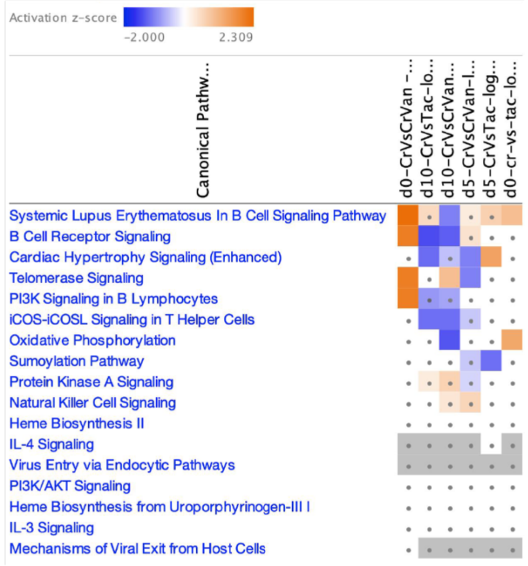

S Figure 6 (Related to Figure 3): Whole blood metatranscriptomics. a Number of quality reads mapped to mouse transcriptome. **b** PCoA plot shows global transcriptome response of mice. **c** Differentially abundant transcripts with log2FC >= 2. D0: day 0 p.i., D5: day 5 p.i.; D10: day 10 p.i., CR+Van: CR treated with vancomycin. **d** Canonical pathway analysis using IPA. gray dots: insignificant pathway (p>=0.1). Blue and orange are enriched in hyperparasitemia resistant and susceptible group, respectively. Column names at the top indicate group comparisons based on day p.i. between susceptible (CR) and resistant group (Tac and CR+Van). Source data are provided in the Source Data file.

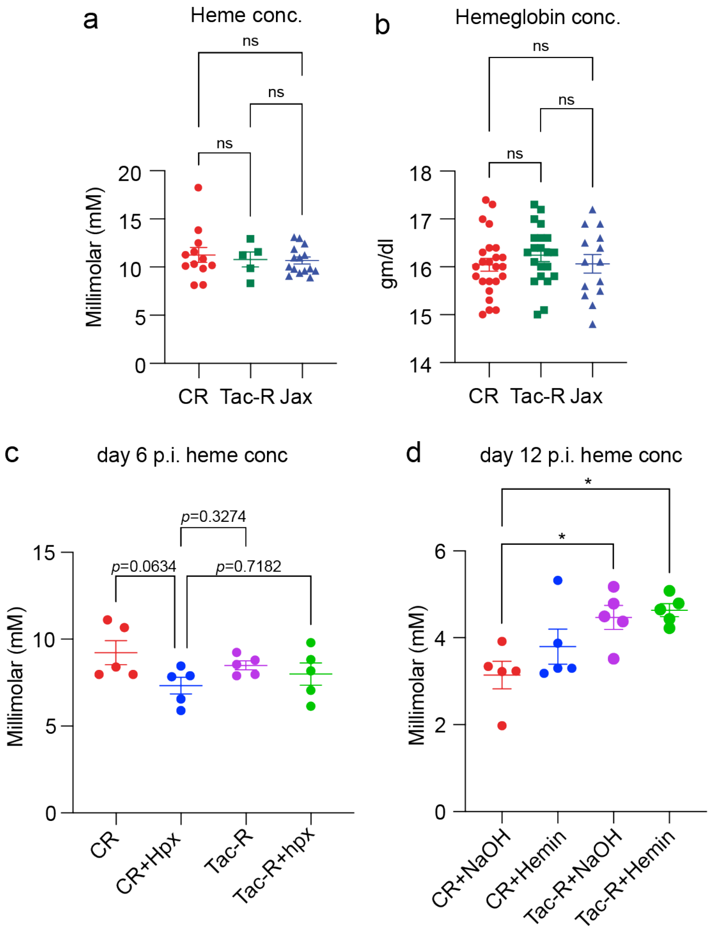

S Figure 7 (Related to Figure 3): Measurement of heme and hemoglobin. a Heme concentration in blood of naïve mice. **b** Hemoglobin concentration in blood of naïve mice. **c** Heme concentration in blood of mice treated with hemopexin on day 6 p.i. **d** Heme concentration in blood of mice treated with Hemin on day 12 p.i. All data are mean ± SE (standard error). Statistical analyses were performed using one-way ANOVA. *$p<0.05$. ns: non-significant. Data in **a** and **b** are from multiple shipments of mice. Heme concentration in **c** and **d** were measured from one experiment.

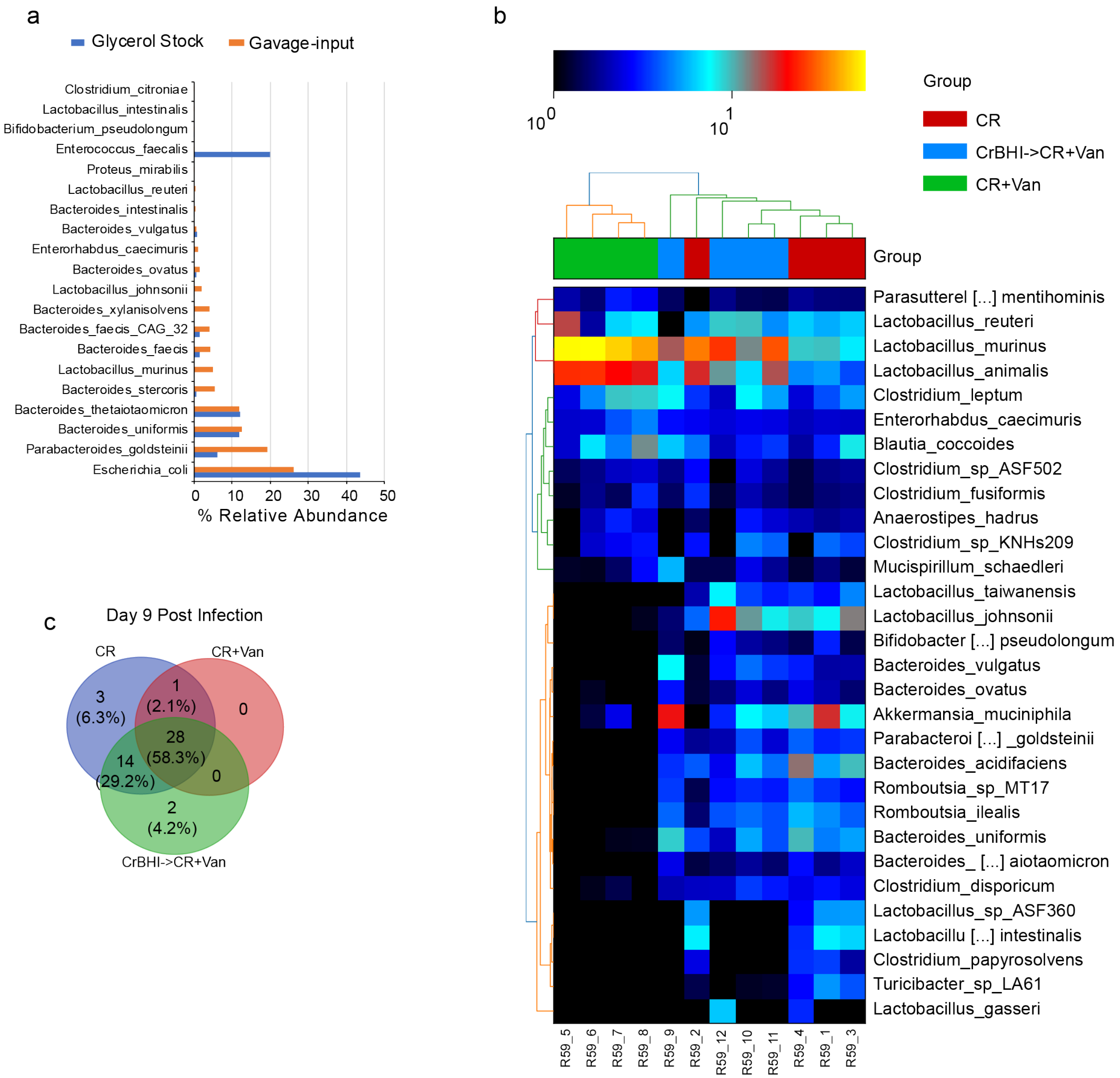

S Figure 8 (Related to Figure 4): Taxonomic profile of CR+Van mice gavaged with CrBHI. a Relative abundance of culturable bacteria of CR ceca grown on BHI agar (CrBHI). Gavage input is the bacterial mass obtained after 48 hrs of incubation in BHI medium using CrBHI glycerol stock as inoculum. Bacterial profiling was performed using shotgun metagenomics. **b** Heatmap shows the abundance of bacterial species on day 9 post Py infection. **c** Venn diagram shows overlap of fecal microbiota on day 9 post Py infection. Source data are provided in the Source Data file.

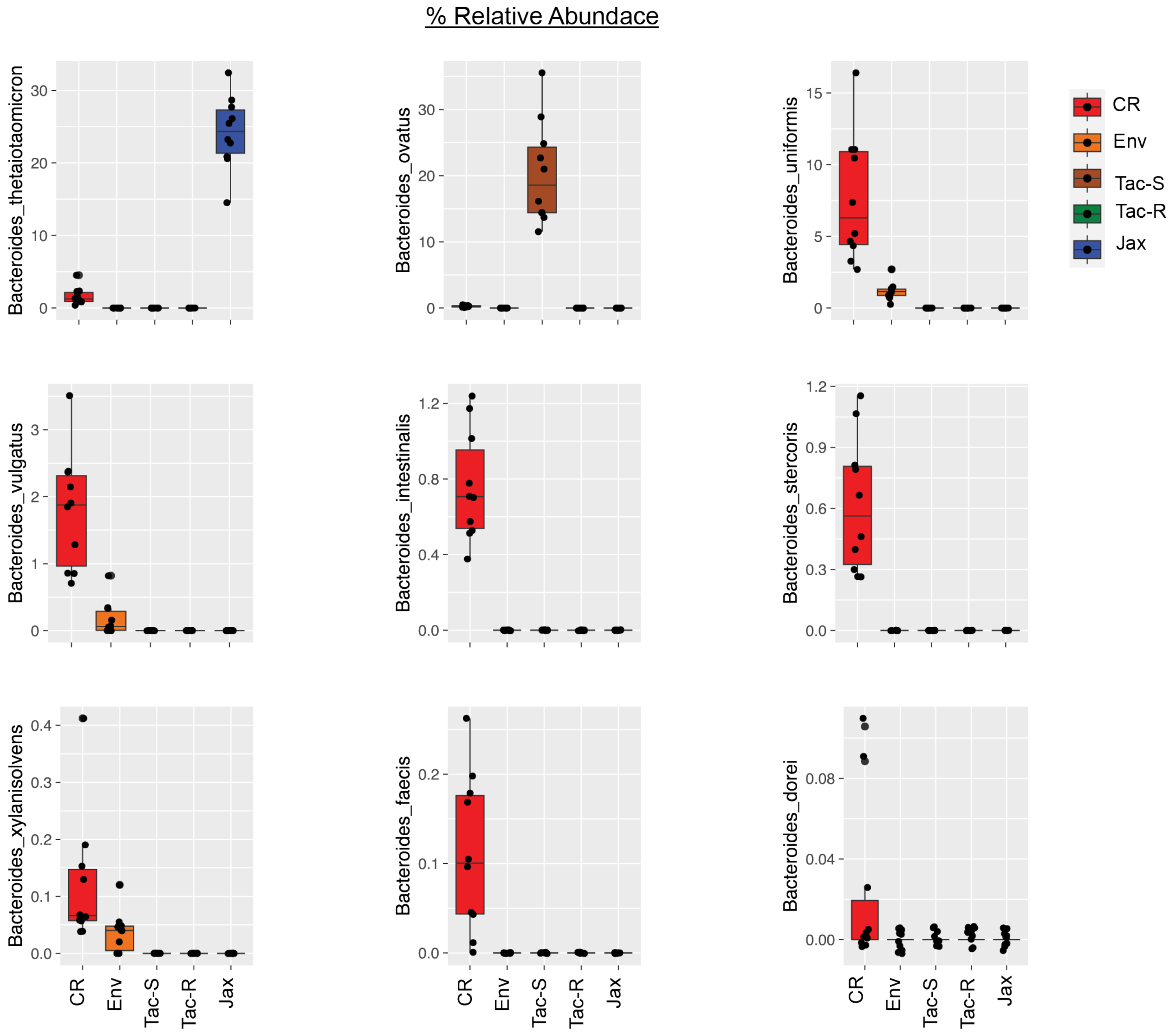

S Figure 9 (Related to Figure 5): Relative abundance (%) *Bacteroides* species detected in ceca of mice using shotgun metagenomics. Relative abundance of bacteria using CLARK pipeline is shown. Lower and upper end of boxplot corresponds to first and third quartile. Horizontal line through the box is median. Data beyond the end of whiskers are outliers. Source data are provided in the Source Data file.

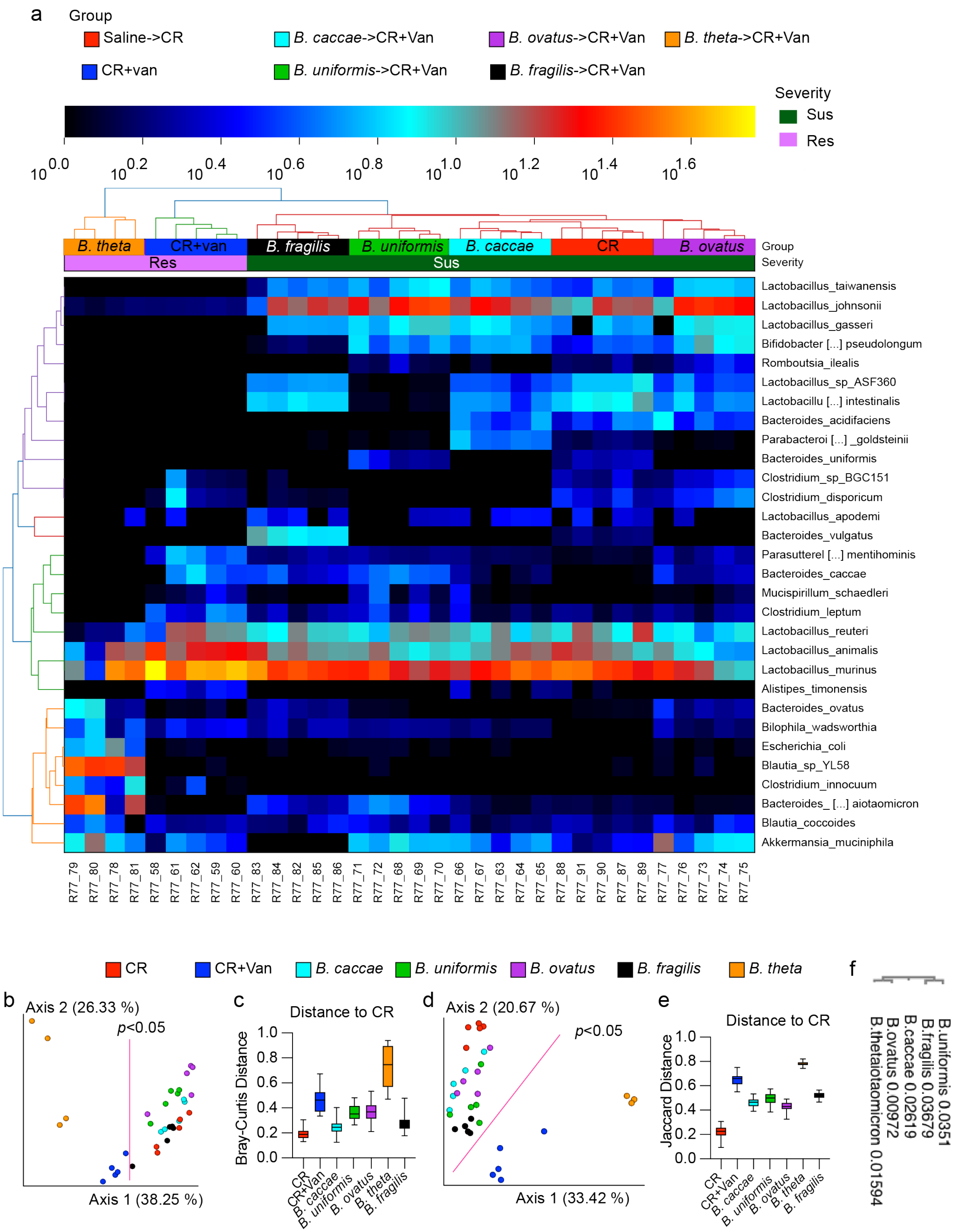

a
Group
Saline->CR
B. caccae->CR+Van
B. ovatus->CR+Van
B. theta->CR+Van
CR+van
B. uniformis->CR+Van
B. fragilis->CR+Van
Severity
Sus
Res
B. theta
CR+van
B. fragilis
B. uniformis
B. caccae
CR
B. ovatus
Lactobacillus_taiwanensis
Lactobacillus_johnsonii
Lactobacillus_gasseri
Bifidobacter [...] pseudolongum
Romboutsia_ilealis
Lactobacillus_sp_ASF360
Lactobacillu [...] intestinalis
Bacteroides_acidifaciens
Parabacteroi [...] _goldsteinii
Bacteroides_uniformis
Clostridium_sp_BGC151
Clostridium_disporicum
Lactobacillus_apodemi
Bacteroides_vulgatus
Parasutterel [...] mentihominis
Bacteroides_caccae
Mucispirillum_schaedleri
Clostridium_leptum
Lactobacillus_reuteri
Lactobacillus_animalis
Lactobacillus_murinus
Alistipes_timonensis
Bacteroides_ovatus
Bilophila_wadsworthia
Escherichia_coli
Blautia_sp_YL58
Clostridium_innocuum
Bacteroides_ [...] aiotaomicron
Blautia_coccoides
Akkermansia_muciniphila
CR
CR+Van
B. caccae
B. uniformis
B. ovatus
B. fragilis
B. theta
b
Axis 2 (26.33 %)
p<0.05
Axis 1 (38.25 %)
c
Distance to CR
Bray-Curtis Distance
d
Axis 2 (20.67 %)
p<0.05
Axis 1 (33.42 %)
e
Distance to CR
Jaccard Distance
f
B.uniformis 0.0351
B.fragilis 0.03679
B.caccae 0.02619
B.ovatus 0.00972
B.thetaiotaomicron 0.01594

S Figure 10 (Related to Figure 5 and 6): Taxonomic profile of CR+Van mice gavaged with individual *Bacteroides* species. Experiments from Figures 5 and 6 were performed with overlapping control (CR) and CR+Van mice. Gut microbiota profiling was done using MVRSION on day 1 p.i. which is 48 hours after the last bacterial gavage. One of the experiments was chosen to profile fecal pellet microbiota. **a** Heatmap shows the abundance of top 30 bacterial species on day 1 Py infection in CR mice treated with vancomycin for two weeks followed by gavage with single *Bacteroides* species. **b** Beta diversity analysis shown by PCoA plot using Bray-Curtis distance of fecal pellet bacteria at species level. **c** Bray-Curtis distance of CR mice to rest of the groups. **d** Beta diversity analysis shown by PCoA plot using Jaccard distance of fecal pellet bacteria at species level. **e** Jaccard distance to CR mice to rest of the groups. **f** Phylogenetic tree based on 16S rRNA gene sequence linked to ATCC strains. This is a neighbor-joining tree without distance corrections using real branch length constructed by Clustal Omega. Line between the graph separates hyperparasitemia susceptible and resistant groups of mice (**b** and **d**). Pairwise PERMANOVA showed all groups were significantly different from each other (**b** and **d**). Box ends indicate first and third quartile. Box plot whiskers represent minimum and maximum value and horizontal line inside the box is mean (**c** and **e**). Source data are provided in the Source Data file.

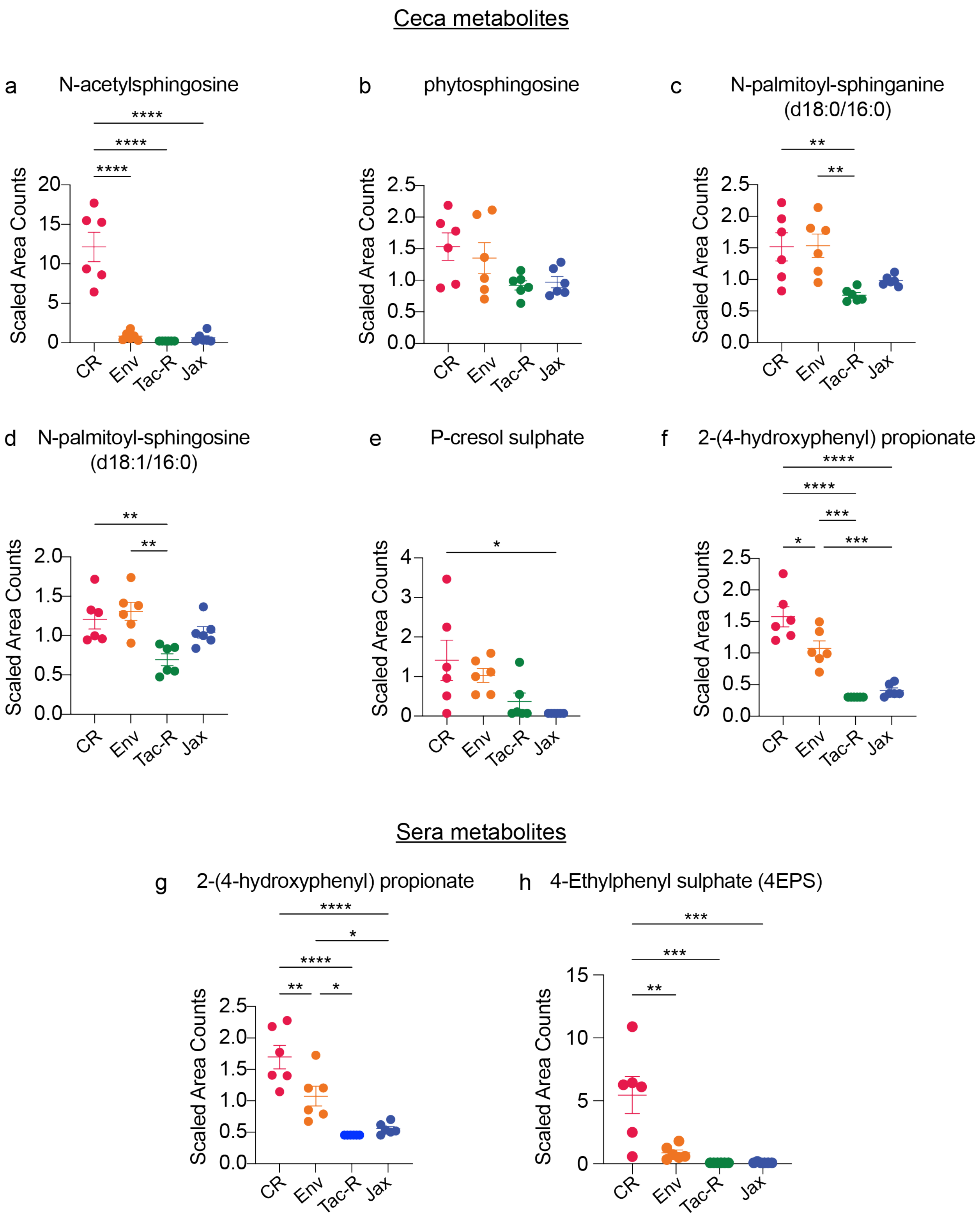

S Figure 11 (Related to Figure 7, S Figure 3 and S Figure 4): Differentially abundant metabolites related to *Bacteroides* genus in ceca and serum of naïve mice. a-f Ceca metabolites. **g** and **h**

Serum metabolites. N=6/group. Data are mean ± SE (standard error). Statistical analyses were performed using ordinary one-way ANOVA. *p<0.05; **p<0.01; ***p<0.001; ****p<0.0001. Source data are provided in the Source Data file.

Supplementary Table 1: Culturable Bacteria identified using MALDI-TOF and sanger sequencing of 16S rRNA.

Colony#	NCBI (refseq_RNA) (16S rRNA)	MALDI-TOF	Media
CrBHI1	Enterococcus faecalis	Enterococcus faecalis	BHI
CrBHI2	Enterococcus faecalis	Enterococcus faecalis	BHI
CrBHI3	Enterococcus faecalis	Enterococcus faecalis	BHI
CrBHI4	Enterococcus faecalis	Enterococcus faecalis	BHI
CrBHI5	Shigella sonnei	Escherichia coli	BHI
CrBHI6	Enterococcus faecalis	Enterococcus faecalis	BHI
CrBHI7	Shigella sonnei	Escherichia coli	BHI
CrBHI8	Enterococcus faecalis	Enterococcus faecalis	BHI
CrBHI9	Enterococcus faecalis	Enterococcus faecalis	BHI
CrBHI10		Enterococcus faecalis	BHI
CrBHI11	Escherichia fergusonii	Escherichia coli	BHI
CrBHI12	Enterococcus faecalis	Enterococcus faecalis	BHI
CrBHI13	Enterococcus faecalis	Enterococcus faecalis	BHI
CrBHI14	Enterococcus faecalis	Enterococcus faecalis	BHI
CrBHI15	Enterococcus faecalis	Enterococcus faecalis	BHI
CrBHI16	Enterococcus faecalis	Enterococcus faecalis	BHI
CrBHI17	Enterococcus faecalis	Enterococcus faecalis	BHI
CrBHI18	Escherichia fergusonii	Escherichia coli	BHI
CrBHI19	Shigella sonnei	Escherichia coli	BHI
CrBHI20	Enterococcus faecalis	Enterococcus faecalis	BHI
CrBHI21	Enterococcus faecalis	Enterococcus faecalis	BHI
CrBHI22	Enterococcus faecalis	Enterococcus faecalis	BHI
CrBHI23	Shigella sonnei	Escherichia coli	BHI
CrBHI24	Shigella sonnei	Escherichia coli	BHI
CrBHI25	Enterococcus faecalis	Enterococcus faecalis	BHI
CrBHI26	Shigella sonnei	Escherichia coli	BHI
CrBHI27	Bacteroides thetaiotaomicron	Escherichia coli	BHI
CrBHI28	Enterococcus faecalis	Enterococcus faecalis	BHI
CrBHI29	Shigella dysenteriae	No organism identification	BHI
CrBHI30		Enterococcus faecalis	BHI
CrBHI31		Enterococcus faecalis	BHI
CrBHI32	Escherichia fergusonii	Escherichia coli	BHI
CrBHI33	Pseudocitrobacter anthropi	Escherichia coli	BHI
CrBHI34		NA	BHI
CrBHI35		Enterococcus faecalis	BHI
CrBHI36	Shigella dysenteriae	Escherichia coli	BHI
CrBHI37	Shigella sonnei	Escherichia coli	BHI
CrBHI38	Shigella sonnei	Escherichia coli	BHI
CrBHI39	Shigella sonnei	NA	BHI
CrBHI40		NA	BHI
CrBHI41	Shigella sonnei	Escherichia coli	BHI
CrBHI42	Escherichia coli	Escherichia coli	BHI
CrBHI43		Enterococcus faecalis	BHI
CrBHI44		Enterococcus faecalis	BHI
CrBHI45	Shigella sonnei	Escherichia coli	BHI
CrBHI46		Enterococcus faecalis	BHI
CrBHI47		Enterococcus faecalis	BHI
CrBHI48	Enterobacter hormaechei	Escherichia coli	BHI
CrBHI49	Shigella sonnei	Escherichia coli	BHI
CrBHI50	Shigella sonnei	Escherichia coli	BHI
CrBHI51		NA	BHI
CrBHI52	Shigella sonnei	Escherichia coli	BHI
CrBHI53		Enterococcus faecalis	BHI
CrBHI54		Escherichia coli	BHI
CrBHI55		Enterococcus faecalis	BHI
CrBHI56		Enterococcus faecalis	BHI
CrBHI57		Lactobacillus murinus	MRS, Ph 5.5
CrBHI58		no peaks found	MRS, Ph 5.5
CrBHI59		no pekas found	MRS, Ph 5.5
CrBHI60		Lactobacillus intestinalis	MRS, Ph 5.5
CrBHI61		no peaks found	MRS, Ph 5.5
CrBHI62		Lactobacillus intestinalis	MRS, Ph 5.5
CrBHI63		Lactobacillus reuteri	MRS, Ph 5.5
CrBHI64		Lactobacillus reuteri	MRS, Ph 5.5
CrBHI65		no peaks found	MRS, Ph 5.5
CrBHI66		No organism identification	MRS, Ph 5.5
CrBHI67		Lactobacillus intestinalis	MRS, Ph 5.5
CrBHI68		Lactobacillus intestinalis	MRS, Ph 5.5
CrBHI69		no peaks found	MRS, Ph 5.5
CrBHI70		Lactobacillus intestinalis	MRS, Ph 5.5
CrBHI71		Lactobacillus intestinalis	MRS, Ph 5.5
CrBHI72		Lactobacillus intestinalis	MRS, Ph 5.5
CrBHI73		Lactobacillus intestinalis	MRS, Ph 5.5
CrBHI74		Lactobacillus intestinalis	MRS, Ph 5.5
CrBHI75		Lactobacillus intestinalis	MRS, Ph 5.5
CrBHI76		Lactobacillus intestinalis	MRS, Ph 5.5
CrBHI77		no peaks found	MRS, Ph 5.5
CrBHI78		Lactobacillus intestinalis	MRS, Ph 5.5
CrBHI79		Lactobacillus intestinalis	MRS, Ph 5.5
CrBHI80		Lactobacillus reuteri	MRS, Ph 5.5
