# Supplementary material for: Gut Bacteroides act in a microbial consortium to cause susceptibility to severe malaria
Source: Nat Commun. 2023 Oct 13;14:6465. doi: 10.1038/s41467-023-42235-0 (PMC10575898; doi:10.1038/s41467-023-42235-0)
Supplement: Supplementary file 3 — Reporting Summary [file 41467_2023_42235_MOESM3_ESM.pdf]

nature portfolio

Corresponding author(s): Nathan W. Schmidt; nwschmid@iu.edu

Last updated by author(s): Aug 7, 2023

Reporting Summary

Nature Portfolio wishes to improve the reproducibility of the work that we publish. This form provides structure for consistency and transparency in reporting. For further information on Nature Portfolio policies, see our Editorial Policies and the Editorial Policy Checklist.

Statistics

For all statistical analyses, confirm that the following items are present in the figure legend, table legend, main text, or Methods section.

n/a	Confirmed	
☐	☒	The exact sample size (*n*) for each experimental group/condition, given as a discrete number and unit of measurement
☐	☒	A statement on whether measurements were taken from distinct samples or whether the same sample was measured repeatedly
☐	☒	The statistical test(s) used AND whether they are one- or two-sided<br>*Only common tests should be described solely by name; describe more complex techniques in the Methods section.*
☐	☒	A description of all covariates tested
☐	☒	A description of any assumptions or corrections, such as tests of normality and adjustment for multiple comparisons
☐	☒	A full description of the statistical parameters including central tendency (e.g. means) or other basic estimates (e.g. regression coefficient) AND variation (e.g. standard deviation) or associated estimates of uncertainty (e.g. confidence intervals)
☐	☒	For null hypothesis testing, the test statistic (e.g. *F*, *t*, *r*) with confidence intervals, effect sizes, degrees of freedom and *P* value noted<br>*Give P values as exact values whenever suitable.*
☒	☐	For Bayesian analysis, information on the choice of priors and Markov chain Monte Carlo settings
☐	☒	For hierarchical and complex designs, identification of the appropriate level for tests and full reporting of outcomes
☐	☒	Estimates of effect sizes (e.g. Cohen's *d*, Pearson's *r*), indicating how they were calculated

Our web collection on statistics for biologists contains articles on many of the points above.

Software and code

Policy information about availability of computer code

Data collection	Microsoft Excel 16.72
Data analysis	Prism 9, FlowJo 10.8.1, Microsoft Excel 16.72, Trimmomatic 0.36, Bowtie 2, samtools 1.5, MetaPhlan2, Clark V1.2.6.1, CZ ID, PhyloSift v1.0.1, QIIME2, HUMAnN2, GhostKoala, Prokka v1.14.5, Megahit v1.2.9, Salmon 1.10.1, MVRSION, Trinity-V2.14.0, IPA v 01-20-04, WebGestalt, MetaboAnalyst 5.0.

For manuscripts utilizing custom algorithms or software that are central to the research but not yet described in published literature, software must be made available to editors and reviewers. We strongly encourage code deposition in a community repository (e.g. GitHub). See the Nature Portfolio guidelines for submitting code & software for further information.

Data

Policy information about availability of data

All manuscripts must include a data availability statement. This statement should provide the following information, where applicable:

- Accession codes, unique identifiers, or web links for publicly available datasets
- A description of any restrictions on data availability
- For clinical datasets or third party data, please ensure that the statement adheres to our policy

All raw sequence are deposited to NCBI Sequence Read Archive (SRA) under the following BioProject: PRJNA962898, PRJNA962885, PRJNA962866, PRJNA962119, PRJNA961982. Raw spectral data from metabolomics study is deposited to EMBL-EBI's MetaboLights under the study: MTBLS3449.

Research involving human participants, their data, or biological material

Policy information about studies with human participants or human data. See also policy information about sex, gender (identity/presentation), and sexual orientation and race, ethnicity and racism.

Reporting on sex and gender	Sex-based analyses was performed in human subjects and not shown to effect malaria outcomes.
Reporting on race, ethnicity, or other socially relevant groupings	Not applicable.
Population characteristics	Healthy community children that were P. falciparum microscopy negative were classified as Pf Neg, while community children that were P. falciparum microscopy positive were classified as Pf pos.<br><br>Severe malarial anemia was defined as P. falciparum smear or RDT positive and serum hemoglobin level ≤ 5 g/dL.
Recruitment	Children between the ages of 0.5 to 4 years old, with 5 most common clinical manifestations of severe malaria (cerebral malaria, CM; respiratory distress, RD; severe malarial anemia, SMA; malaria with complicated seizures, M/S; and prostration) were enrolled in a prospective longitudinal cohort study at two sites: 1) the Pediatric Acute Care Unit at Mulago National Referral Hospital in Kampala, Uganda, and 2) the Pediatric Emergency Ward at the Jinja Regional Referral Hospital in Jinja, Uganda. Children from the same neighborhoods without active illness or fever at time of enrollment were enrolled in the healthy community control group.
Ethics oversight	Ethics approval and consent to participate Written informed consent was obtained from the parents or legal guardians of all study participants. Ethical approval was granted by the Institutional Review Boards at Makerere University School of Medicine (Ref: 2013-141, date approved: September 23, 2014), the University of Minnesota (Ref: 1309M42501, date approved: September 23, 2013) and subsequently moved to Indiana University (Ref: 1412213778, date approved: January 20, 2015). The Uganda National Council for Science and Technology approved the study (Ref: HS1522, date approved: May 12, 2013)

Note that full information on the approval of the study protocol must also be provided in the manuscript.

Field-specific reporting

Please select the one below that is the best fit for your research. If you are not sure, read the appropriate sections before making your selection.

- [x] Life sciences
- [ ] Behavioural & social sciences
- [ ] Ecological, evolutionary & environmental sciences

For a reference copy of the document with all sections, see nature.com/documents/nr-reporting-summary-flat.pdf

Life sciences study design

All studies must disclose on these points even when the disclosure is negative.

Sample size	Stool sample size was determined based on the number of available stool samples from children in the different categories at time of study initiation.<br><br>Sample for mouse studies were calculated with five mice/group based on experience with a range of experimental designs and readouts studying murine malaria and the gut microbiome that yields >80% power when alpha equals 0.05 to identify as little as 1.6-fold differences with standard deviations ranging 20-40% of the group means.
Data exclusions	No data were excluded
Replication	Mouse experiments have been replicated at least twice. Analysis of stool bacteria community analysis has not been replicated.
Randomization	Children were assigned to respective groups based on clinical definitions of those groups.
Blinding	Investigators were not blinded owing to the need treat different groups of mice, and monitor outcomes and the nature of the clinical observation study.

Reporting for specific materials, systems and methods

We require information from authors about some types of materials, experimental systems and methods used in many studies. Here, indicate whether each material, system or method listed is relevant to your study. If you are not sure if a list item applies to your research, read the appropriate section before selecting a response.

Materials & experimental systems

n/a	Involved in the study	
☐	☒	Antibodies
☒	☐	Eukaryotic cell lines
☒	☐	Palaeontology and archaeology
☐	☒	Animals and other organisms
☐	☒	Clinical data
☒	☐	Dual use research of concern
☒	☐	Plants

Methods

n/a	Involved in the study	
☒	☐	ChIP-seq
☐	☒	Flow cytometry
☒	☐	MRI-based neuroimaging

Antibodies

Antibodies used	CD45.2-APC, Clone 104 Biolegend Cat# 109814; Ter119-APC/Cy7, Clone TER-119 Biolegend Cat# 116223.
Validation	Antibodies were validated by the manufacturing company. Antibody staining was comparable between production lots as observed in the Schmidt laboratory.

Animals and other research organisms

Policy information about studies involving animals; ARRIVE guidelines recommended for reporting animal research, and Sex and Gender in Research

Laboratory animals	Six-weeks old female C57BL/6 mice were purchased from four different vendors: Charles River Laboratories (CR), Taconic Biosciences (Tac), Jackson Laboratories (Jax), and Envigo (Env). Mice were obtained from CR Isolated Barrier Unit (IBU) R01; Py hyperparasitemia-susceptible Tac mice (Tac-S) from IBU 001501C and Py hyperparasitemia-resistant Tac mice (Tac-R) from IBU 050401C; Env mice from IBU 202A; and Jax from IBU JAXEast:AX4. Mice were housed in a specific pathogen-free (SPF) facility, kept on irradiated NIH-31 Modified Open Formula Mouse/Rat diet (#7913; Envigo, Indianapolis, IN), and provided non-acidified autoclaved reverse osmosis water ad libitum. Mice were housed in a 12-hour light (6 AM – 6 PM) and 12-hour dark (6 PM – 6 AM) cycle at ambient air temperature (about 22 degrees C). Mice were acclimatized for one week before treatments.
Wild animals	No wild animals were used.
Reporting on sex	Sex was not evaluated in this study design.
Field-collected samples	No field collected samples were used.
Ethics oversight	Animal procedures and experiments were approved by Institutional Animal Care and Use Committees (IACUC) from the University of Louisville and Indiana University.

Note that full information on the approval of the study protocol must also be provided in the manuscript.

Clinical data

Policy information about clinical studies

All manuscripts should comply with the ICMJEguidelines for publication of clinical research and a completedCONSORT checklist must be included with all submissions.

Clinical trial registration	Not applicable.
Study protocol	The human subject protocol is available at Indiana University
Data collection	Children were enrolled in a prospective longitudinal cohort study at two sites: 1) the Pediatric Acute Care Unit at Mulago National Referral Hospital in Kampala, Uganda, and 2) the Pediatric Emergency Ward at the Jinja Regional Referral Hospital in Jinja, Uganda.
Outcomes	No applicable.

Flow Cytometry

Plots

Confirm that:

- [x] The axis labels state the marker and fluorochrome used (e.g. CD4-FITC).
- [x] The axis scales are clearly visible. Include numbers along axes only for bottom left plot of group (a 'group' is an analysis of identical markers).
- [x] All plots are contour plots with outliers or pseudocolor plots.
- [x] A numerical value for number of cells or percentage (with statistics) is provided.

Methodology

Sample preparation	About 5 ul whole blood was collected from a tail snip in 100 ul cold 1X PBS. Whole blood was fixed in 0.00625% glutaraldehyde, stained with conjugated antibodies, and ran immediately on a flow cytometer.
Instrument	Attune Next (Invitrogen) and BD LSRFortessa (BD Bioscience)
Software	FlowJo 10.8.1
Cell population abundance	Not applicable.
Gating strategy	Has been reported in previous publications from multiple laboratories.

- [ ] Tick this box to confirm that a figure exemplifying the gating strategy is provided in the Supplementary Information.
